# Stress and Pain Before, During and After the First Wave of the COVID-19 Pandemic: An Exploratory Longitudinal Mixed Methods Study

**DOI:** 10.3389/fpain.2021.725893

**Published:** 2021-11-24

**Authors:** M. Gabrielle Pagé, Lise Dassieu, Élise Develay, Mathieu Roy, Étienne Vachon-Presseau, Sonia Lupien, Pierre Rainville

**Affiliations:** ^1^Department of Anesthesiology and Pain Medicine, Faculty of Medicine, Université de Montréal, Montreal, QC, Canada; ^2^Department of Psychology, Faculty of Arts and Science, Université de Montréal, Montreal, QC, Canada; ^3^Research Center of the Centre hospitalier de l'Université de Montréal (CRCHUM), Montreal, QC, Canada; ^4^Department of Psychology, Faculty of Science, McGill University, Montreal, QC, Canada; ^5^Alan Edwards Centre for Research on Pain, McGill University, Montreal, QC, Canada; ^6^Faculty of Dentistry, McGill University, Montreal, QC, Canada; ^7^Department of Anesthesia, Faculty of Medicine, McGill University, Montreal, QC, Canada; ^8^Centre for Studies on Human Stress, Montreal Mental Health University Institute, Montreal, QC, Canada; ^9^Department of Psychiatry, Faculty of Medicine, University of Montreal, Montreal, QC, Canada; ^10^Centre de recherche de l'Institut universitaire de gériatrie de Montréal, CIUSSS Centre-sud-de l'île de Montréal, Montreal, QC, Canada; ^11^Department of Stomatology, Université de Montréal, Montreal, QC, Canada

**Keywords:** chronic pain (MeSH), stress, COVID-19, pandemic, mixed methods, control, unpredictability

## Abstract

**Aims:** This study explores the association between subjective feeling of stress and pain experience in the context of the COVID-19 pandemic with a focus on characteristics known to trigger a physiological stress response [sense of low control, threat to ego, unpredictability and novelty (STUN)].

**Methods:** This exploratory longitudinal convergent mixed methods design consisted of online questionnaires over three time points (before, during and after the 1st wave of the COVID-19 pandemic) (*N* = 49) and qualitative interviews (*N* = 27) during the 1st wave of the pandemic on distinct samples of individuals living with chronic pain (CP). Both types of data sources were mixed upon integration using joint display.

**Results:** Mean pain intensity scores remained stable across time points, while pain unpleasantness and pain interference scores significantly improved. Global impression of change scores measured during the first wave of the pandemic do not entirely concord with pain scores evolution. Two thirds of participants reported a global deterioration of their pain condition at the beginning of the pandemic. Stress and pain catastrophizing before the pandemic were associated with pain scores throughout the pandemic; while most specific measures of stress due to the novel, uncontrollable, unpredictable and threatening nature of the pandemic were not. Qualitative data demonstrated that the deterioration reported in pain status reflected additional dimensions, including spatial expansion of the painful area, reduced access to treatments and challenges in adapting pain management strategies.

**Conclusions:** Helping individuals to negotiate stressful aspects of the pandemic might help offset the negative impacts of stress on pain status in this context or other important life events.

## Introduction

The SARS-CoV-2 was identified in January 2020 as the cause of the coronavirus disease 2019 (COVID-19). Since then, this pandemic has been associated with more than 3 million deaths and 235 million confirmed cases as of October 7th 2021, more than 20 months after the first case was detected (1). In the province of Quebec, Canada, almost 1,000 cases and 150 deaths due to COVID-19 were reported daily during the first wave, for a population of 8.1 million inhabitants. The province of Quebec enforced lockdown of schools, office buildings, sports installations, restaurants, shopping malls in addition to postponing most non-urgent medical appointments. Notably, reopening was announced and postponed several times, until the end of May. The Quebec context during that specific time offered a unique opportunity to study the interaction between stress and chronic pain.

These effects constitute potential sources of stress that might have a particularly devastating impact on individuals living with chronic pain (2). Pain might deteriorate during the COVID-19 lockdown because of the direct impact of stress on pain (3, 4), or through indirect effects such as unpredictable access to pain care and management facilities, increased social isolation, and poor sleep (2, 5–10).

A multitude of studies have documented the complex associations between stress and pain, varying from stress-induced analgesia to stress-induced hyperalgesia (11–14). Furthermore, stress has also been identified as an important factor that could increase risks of comorbid psychological distress such as depression in this population (15, 16). Not every individual react the same way to sources of stress however, and understanding how individual appraisal of the threat and challenges posed by a new stressor such as the pandemic, as well as identifying vulnerability and resilience factors can help better understand the experience of individuals and its impact on pain evolution and its management (17, 18).

To better understand stress reactions, it is necessary to understand what stress is and how it triggers a physiological response (body's response to the detection of a threat—i.e., secretion of cortisol, noradrenaline). Decades of research have shown that when individuals perceive being in a situation over which they have a sense of low control (S), that poses a social-evaluative threat (T), is unpredictable (U) and/or is novel (N)—[thereby referred to the STUN characteristics], this will activate the hypothalamic-pituitary-adrenal axis and produce a stress response (19–21). The STUN framework appears to be an interesting and comprehensive approach to understanding the associations between stress and chronic pain, especially considering that they are not traditionally explored together within a comprehensive framework (22). Being able to characterize individuals' pain, stress and psychological characteristics and understand how these factors change once they are simultaneously exposed to a world-wide outbreak presents a unique opportunity to further our understanding of how and for whom stress has a significant impact on pain and psychological distress.

## Objectives

The overall study goal was to explore the evolution of pain experiences among individuals living with chronic pain before and during the first wave of the COVID-19 pandemic in the province of Quebec, Canada, and understand how individual appraisal of the threats and challenges posed by the pandemic, influence this evolution. The specific quantitative (Study 1), qualitative (Study 2) and mixed methods (MM) goals were as follow:

*Study 1—Quantitative examination:*

(1) Examine the evolution of pain intensity, unpleasantness and interference scores at baseline, during and after the first wave of the pandemic.
(2) Document individuals' perceived global impression of change in pain status, and psychological distress during and after the first wave of the COVID-19 pandemic.
(3) Identify pre-pandemic stress-related indices (STUN characteristics, global perceived stress and pain catastrophizing) associated with the evolution of pain and psychological distress (anxiety and depressive symptoms) across the first wave of the COVID-19 pandemic.

*Study 2 – Qualitative inquiry:*

(4) Explore the dynamic impact of stress on the pain experience during the first wave of the COVID-19 pandemic from the perspective of people with chronic pain; and

*Mixed Methods Integration:*

(5) Obtain a more comprehensive understanding of the relationship between stress and pain experience during the COVID-19 pandemic by exploring convergence and divergence of the quantitative and qualitative findings.

The purpose of mixed methods in this study was thus to provide complementary and more comprehensive views of the phenomena under study and to take into account the diversity of perspectives on the experience (23).

## Overall Study Design

**Figure 1:**
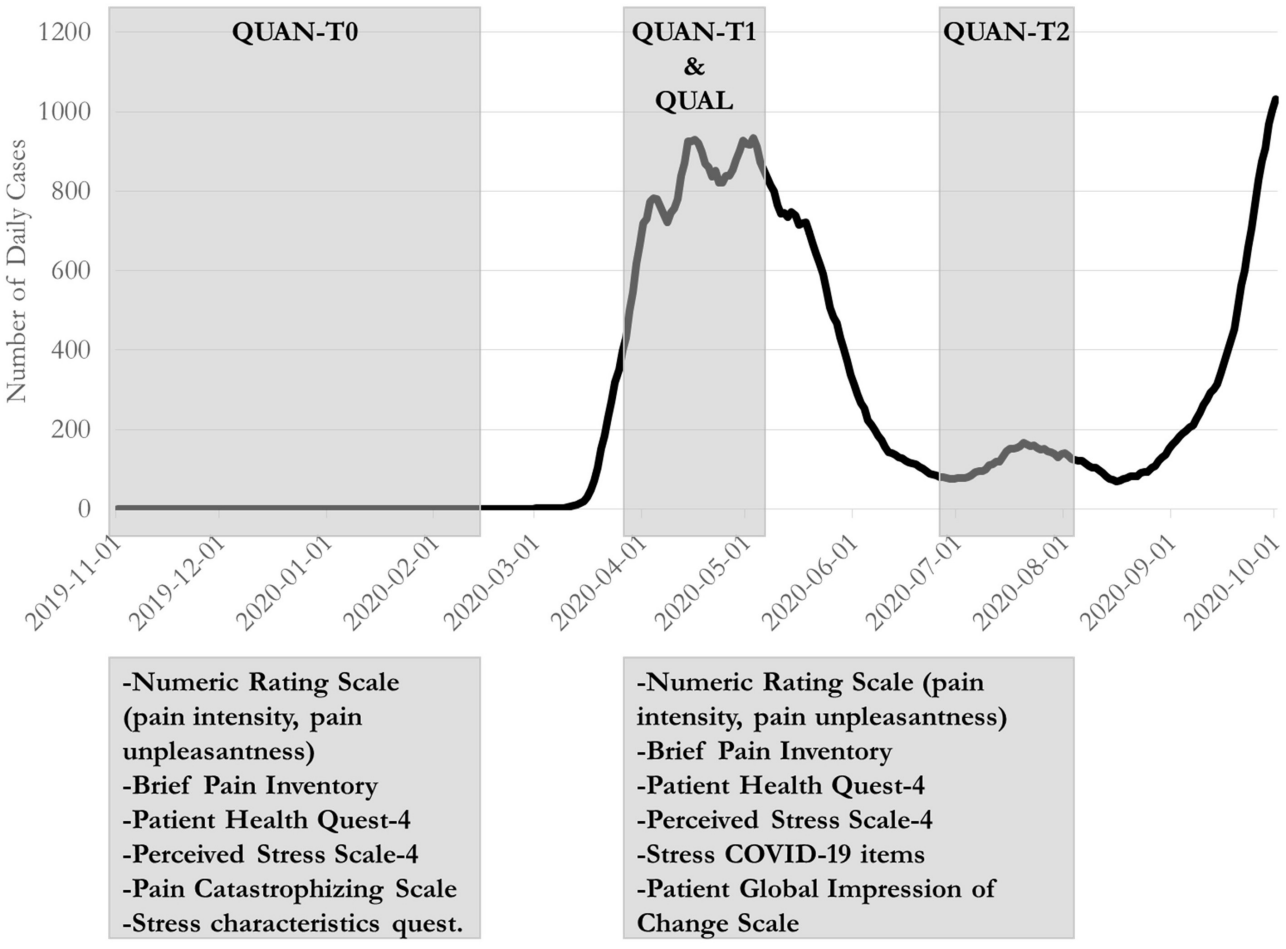
Timing of baseline and follow-up measures in relation to the evolution of the COVID-19 pandemic in Quebec, Canada. The shaded gray zones refer to the periods during which participants completed the time point measures or interviews.

This study adopted a longitudinal convergent design with triangulation in which quantitative (Study 1) and qualitative (Study 2) data were collected in parallel using different samples and integrated using previously described methods (24, 25). Figure 1 shows the timeline of the QUAN and QUAL studies, overlapping with the progression of the COVID-19 pandemic.

The study was approved by the research ethics board of the *Center hospitalier de l' Université de Montréal* (18.368-YP) and written consent was obtained from study participants. Here we first present the methodology, results and brief discussion of Study 1 and Study 2 separately, and finally the methods and results of the mixed methods integration. Samples were independent for Study 1 and Study 2.

## Study 1—Quantitative Stress and Pain Investigation

### Materials and Methods

#### Study Design

The quantitative study adopted a longitudinal, prospective study design with three distinct time points: T0 (before the pandemic), T1 (during the first wave of the pandemic), and T2 (after the first wave of the pandemic).

#### Participants

Participants were initially recruited through an ad sent electronically in November 2019 to all members (approximately 9,000 individuals) of a community-based organization for individuals living with chronic pain for one of three studies exploring the associations between stress characteristics and chronic pain. Out of more than 600 individuals who manifested interest in the study, 54 were enrolled in this particular project until February 2020. At that time enrollment stopped because of the potentially confounding impact of the pandemic. When COVID-19 pandemic began and after obtaining ethics approval, participants who had already completed the study were solicited to participate in additional follow-up measurements to capture the impact of the pandemic on stress and pain. Eligibility criteria were assessed by phone and included having non-cancer pain of more than 3 months duration and of moderate to severe intensity (>3 on a 0–10 point scale), living in the province of Quebec, being fluent in written and spoken French, being aged 18 years or older, and having access to the Internet. Participants were excluded if they had a cognitive or physical impairment that made it impossible to complete self-reported questionnaires.

#### Procedures

*Baseline (T0)*: After providing written consent electronically, participants completed an online battery of questionnaires documenting their overall stress, pain-related stress, pain characteristics and quality of life. They also completed a 1-week electronic diary that aimed to explore optimal methodological approaches to collect daily information on stress and pain, but the results are not presented as part of this study.

*T1 and T2*: During the first wave of the COVID-19 pandemic, all participants were re-contacted and invited to participate in a follow-up study to re-examine the associations between stress and pain during the pandemic. Forty-nine out of the 54 participants agreed to participate in these additional time points [during (T1) and after (T2) the first wave of the pandemic]. They followed the same procedure established for the baseline assessment to document stress related to the pandemic, overall stress, and pain characteristics using online questionnaires and electronic diary. Participants were compensated a total of $60 for the study.

#### Measures

Measures were selected to assess general and pain-specific stress and psychological responses to pain that might influence pain and psychological distress during the pandemic.

The following measures were administered across all three time points:

The *Brief Pain Inventory* [BPI (26)] is a measure of the impact of pain on daily function, pain location, pain medication, and amount of pain relief over 24-h period. Seven items, each rated on a 0–10 scale, document the extent to which pain impacts on daily function. This composite score had good reliability and validity in various chronic pain populations (27). In this study, α = 0.78–0.85 at T0–T2.

The *Patient Health Questionnaire-4* [PHQ-4 (28)] is a brief measure of psychological distress with the following classification: normal (0–2), mild (3–5), moderate (6–8) and severe (9–12). Two items are drawn from the Patient Health Questionnaire-9 and evaluates depressive symptoms and two items are drawn from the Generalized Anxiety Disorder-7 scale and evaluates anxious symptoms. The PHQ-4 had good validity and adequate reliability (28). In this study, α = 0.70–0.78 at T0–T2.

The *Perceived Stress Scale-4* [PSS4 (29)] is a 4-item self-reported measure that assesses the extent to which individuals perceive their life as being unpredictable, uncontrollable and overloaded over the previous month. The scale had excellent validity and internal consistency (29). In this study, α = 0.74–0.83 at T0–T2.

The following measures were administered at baseline only:

The *Pain Catastrophizing Scale* [PCS (30)] measures the extent to which individuals ruminate, feel helpless, and magnify their pain experience. Each item is rated on a scale from 0 to 4, and items are summed to create a total score that ranges from 0 to 52. The PCS has been shown to have adequate internal consistency, reliability and sensitivity to change over time (31). In this study, α = 0.93 at T0.

The *Stress Characteristics Questionnaire* [SCQ; (32)] measures one's sensitivity to each of the four characteristics associated with a physiological response to stress (19), namely Sense of low control, Threat to ego (one's personality), Unpredictability, and Novelty. Each dimension is measured by summing 5 Likert-type items that ask participants to rate on a scale from 0 (not stressful at all) to 10 (extremely stressful) the extent to which they would find each situation described as stressful. Higher scores indicate higher stress responsivity. In this study, α = 0.64 for control subscale, α = 0.72 for the ego subscale, α = 0.76 for unpredictability subscale, and α = 0.80 for the novelty subscale at T0. The psychometric properties of the original questionnaire have not yet been published. As such, the validity of the questionnaire is unknown.

Additional *pain characteristics* were measured, including pain duration. Pain intensity (mean, and worst pain intensity) and pain unpleasantness over the past 7 days were assessed using a Numeric Rating Scale (33, 34) (NRS, duration).

The following measures were administered at T1 and T2:

A series of questions on *Stress related to the COVID-19 pandemic* were administered on a 0–10 scale to assess the extent to which individuals found the pandemic to be stressful. Two questions aimed to measure overall stress related to the pandemic: “To what extent do you find the COVID-19 pandemic stressful,” and “To what extent do you find the lockdown measures associated with the COVID-19 pandemic stressful”. Four questions aimed to measure the four dimensions of the STUN model (Ego: “My behaviors and emotions about the COVID-19 pandemic have a negative impact on the opinion I have of myself;” Control: “The feeling of having no control over the evolution of the pandemic causes me stress,” Novelty: The novelty of the current pandemic causes me stress;” Unpredictability: “The unpredictable evolution of the pandemic causes me stress”). These questions were developed by expert consensus (i.e., authors and other researchers with expertise in pain and/or stress research) at the beginning of the pandemic.

The *Patient Global Impression of Change scale* [PGIC (35)] was administered to document whether participants perceived a change in their pain status since the beginning of the pandemic on a scale ranging from 1 (completely deteriorated) to 7 (completely improved); a score between 1 and 3 indicates some deterioration; a score of 4 indicates no change and a score above 4 indicates some improvement. An open-ended question was also included that asked participants to describe how and why their pain status had changed. The PGIC has been recommended by the Initiative on Methods, Measurement and Pain Assessment in Clinical Trials (IMMPACT) group (33) and has been shown to mediate individual differences in a number of chronic pain outcomes associated with expectations (36).

#### Data Analysis

Descriptive statistics (frequencies, percentages, means and standard errors) were used to characterize the study sample and follow the evolution of pain and stress over time.

*Objective 1 examined the evolution of pain and psychological distress across the first wave of the pandemic using linear mixed effect analysis*. Models included a linear and a quadratic time trend, and a random effect of participant with a restricted maximum likelihood estimation used to determine whether scores on pain intensity (NRS pain intensity), pain unpleasantness (NRS pain unpleasantness), pain interference (BPI) or psychological distress (PHQ-4 total score) significantly changed across time. If quadratic term was significant it was retained in the model for Obj. 2, otherwise it was not included. Box plots were also used to compare changes in NRS pain intensity scores between T0 and T1, and between T1 and T2 according to participants' global impression of change in their pain status at T1 and T2, respectively.

*Objective 2 examined characteristics associated with evolution of pain and psychological distress using linear mixed effect analysis*. Models were used to identify baseline stress-related characteristics (pain catastrophizing, perceived stress (PSS-4) and scores on each of the four dimensions of the SCQ) associated with (a) pain intensity (NRS pain intensity), (b) pain interference (BPI), and (c) psychological distress (PHQ-4) across the first wave of the pandemic. Intercept was included as a random effect.

Alpha was set at 0.05. No correction was applied for multiple comparisons given that it further contributes to reducing statistical power, increases risks of Type II errors, and contributes to negative publication bias (37). Information regarding the clinical meaningfulness of statistically significant results is provided when relevant. Sensibility analyses were also conducted to examine the unique contribution of the SCQ variables alone. The linear mixed effect models were thus re-run to exclude the PCS and PSS.

#### Results

Out of 54 individuals initially recruited, 49 completed at least one of the follow-ups and thus were included in the quantitative analyses. Participants' characteristics are shown in Table 1. Three quarters of participants identified as female (*n* = 36; 75.0%). More than half of participants (*n* = 26; 57.1%) were not working due to disability and the average pain duration was 15.11 years (sd = 11.3; min = 2, max = 43). Average pain intensity scores varied by <10% across the three time points, which is considered to be below what is considered as clinically meaningful (38).

**Table 1:** Socio-demographic, pain, stress and psychological characteristics of individuals living with chronic pain before, during and after the first wave of the COVID-19 pandemic.

	**Study time points relative to the 1st wave of the COVID-19 pandemic**		
	**Before (*n* = 49)**	**During (*n* = 48)**	**After (*n* = 46)**
**Socio-demographic characteristics**			
Gender (*N* [%])		–	–
Woman	36 [75.0]		
Man	12 [25.0]		
Missing	1		
Age mean ± sd	51.13 ± 10.6	–	–
Min	30		
Max	78		
Living Environment (*N* [%])		–	–
Rural	9 [19.6]		
Urban	37 [80.4]		
Missing	3		
Race (*N* [%])			
White	44 [89.8%]		
Prefer not to answer/missing data	5 [10.2%]		
Education level (*N* [%])		–	–
High school	7 [14.3]		
Technical degree	26 [53.0]		
University	16 [32.7]		
Living condition (*N* [%])		–	–
Alone	12 [25.0]		
Family members	36 [75.0]		
Missing	1		
Work status (*N* [%])		–	–
Working	16 [28.6]		
Invalidity	26 [57.1]		
Retired	7 [14.3]		
Work status change (*N* [%])	–		
Same as pre-pandemic		5 [10.4]	10 [21.7]
Temporarily laid-off		5 [10.4]	3 [6.5]
Remote working		6 [12.5]	3 [6.5]
Not applicable		28 [58.4]	28 [60.9]
Missing		4 [8.3]	2 [4.4]
**Psychological and stress characteristics**			
Pain catastrophizing (PCS)	20.35 ± 11.8		
Stress characteristics based on the STUN framework (SCQ) (0-50)		–	–
Sense of low control	24.24 ± 10.4		
Threat to ego	27.08 ± 10.1		
Unpredictability	25.67 ± 10.6		
Novelty	31.10 ± 8.3		
Perceived stress scale (PSS-4)	7.41 ± 3.1	7.10 ± 2.4	7.48 ± 3.1
Psychological distress (PHQ-4)			
None-mild (0–5)	31 [63.3]	28 [58.3]	31 [67.4]
Moderate-severe (6–12)	18 [36.7]	20 [41.7]	15 [32.6]
Stress associated with COVID-19 pandemic (0–10)	–	7.16 ± 2.4	6.72 ± 2.4
Stress associated with lockdown measures (0–10)	–	5.86 ± 28.1	5.03 ± 2.6
Stress associated with (0–10):	–		
Sense of low control related to pandemic		5.67 ± 3.0	4.76 ± 2.9
Threat to the ego related to pandemic		2.48 ± 2.6	2.41 ± 2.8
Unpredictability of pandemic		6.48 ± 2.7	5.89 ± 2.5
Novelty of pandemic		5.69 ± 2.9	5.26 ± 2.6
**Pain and health-related characteristics**			
Pain duration (years)	15.11 ± 11.3	–	–
Mean Pain Intensity (NRS-11)	5.86 ± 1.4	6.08 ± 2.0	5.63 ± 1.8
Worst Pain Intensity (NRS-11)	8.16 ± 1.3	8.06 ± 1.5	7.70 ± 1.8
Pain Unpleasantness (NRS-11)	7.33 ± 1.8	6.42 ± 2.4	6.35 ± 2.2
Pain Interference (BPI)	5.90 ± 1.8	5.11 ± 2.1	5.09 ± 2.1
Global impression of change—pain status (*N* [%])	–		
Considerably deteriorated		2 [4.2]	3 [6.5]
Moderately deteriorated		9 [18.8]	4 [8.7]
Slightly deteriorated		21 [43.8]	15[32.6]
Unchanged		13 [27.1]	19[41.3]
Slightly improved		3 [6.3]	3[6.5]
Moderately improved		0 [0.0]	0[0.0]
Greatly improved		0 [0.0]	2[4.3]
Reason for pain deterioration (*N* [%])	–		
Increased stress		20 [62.5]	9 [40.9]
Delayed pain treatments		5 [15.6]	5 [22.7]
Other		6 [18.8]	6 [27.3]
Missing		1 [3.1]	2 [9.1]

*The statistics are represented as mean ± sd unless otherwise specified*.

*PCS, Pain Catastrophizing Scale; SAM-S-P, Stress Appraisal Measure-Stressfulness subscale applied to pain; PSS-4, Perceived Stress Scale-short version; SCQ, Stress Characteristics Questionnaire; NRS-11, 0-10 Numeric Rating Scale; BPI, Brief Pain Inventory; QofL, Quality of Life*.

*Obj 1. Evolution of Pain and Psychological Distress*. There were no significant linear or quadratic effect of time for pain intensity or psychological distress across the first wave of the pandemic (*p* > 0.05). There were significant linear (β = −1.93, *p* = 0.010) and quadratic (β = 0.37, *p* = 0.042) effects of time on levels of pain interference (BPI). Finally, there was a significant linear effect of time for pain unpleasantness (β = −2.17, *p* = 0.036). Results of the linear mixed effects models are shown in Table 2.

**Table 2:** Linear mixed effects models examining the within-person evolution of pain and psychological distress (*N* = 49).

**Fixed effects**	**β**	**SE**	***t***	***p***
**Pain intensity**				
Intercept	5.02	0.63	7.99	<0.001
Time	1.16	0.68	1.70	0.091
Time^2^	−0.33	0.17	−1.04	0.054
**Pain unpleasantness**				
Intercept	9.08	0.91	9.93	<0.001
**Time**	**−2.17**	**1.01**	**−2.15**	**0.036**
Time^2^	0.41	0.25	1.65	0.105
**Pain interference (BPI)**				
Intercept	7.46	0.68	10.98	<0.001
**Time**	**−1.93**	**0.72**	**−2.67**	**0.010**
**Time** ^**2**^	**0.37**	**0.18**	**2.09**	**0.042**
**Psychological distress (PHQ)**				
Intercept	4.53	1.44	3.15	0.003
Time	0.62	1.61	0.39	0.700
Time^2^	−0.18	0.40	−0.44	0.659

*B, unstandardized regression coefficients; SE, standard error; BPI, Brief Pain Inventory; PHQ, Patient Health Questionnaire-4. Bold values indicate p < 0.05*.

As shown in Table 1, 32 participants reported deteriorated pain during the first wave of the COVID-19 pandemic using the Patient Global Impression of Change Scale. Reasons reported by participants for this deterioration included stress (*n* = 19/32; 59.8%), postponed pain treatments (*n* = 5/32; 15.6%), ergonomic issues associated with working from home (*n* = 2/32; 6.3%), and sleep difficulties (*n* = 2/32; 6.3%). Thirteen reported unchanged pain. Only three participants reported improved pain that they attributed to a slower pace during the pandemic (e.g., less scheduled activities, not having to commute to work). Twenty-two participants (out of 46; 47.8%) reported that their pain deteriorated after the first wave (T2) compared to during the first wave (T1) of the pandemic.

**Figure 2:**
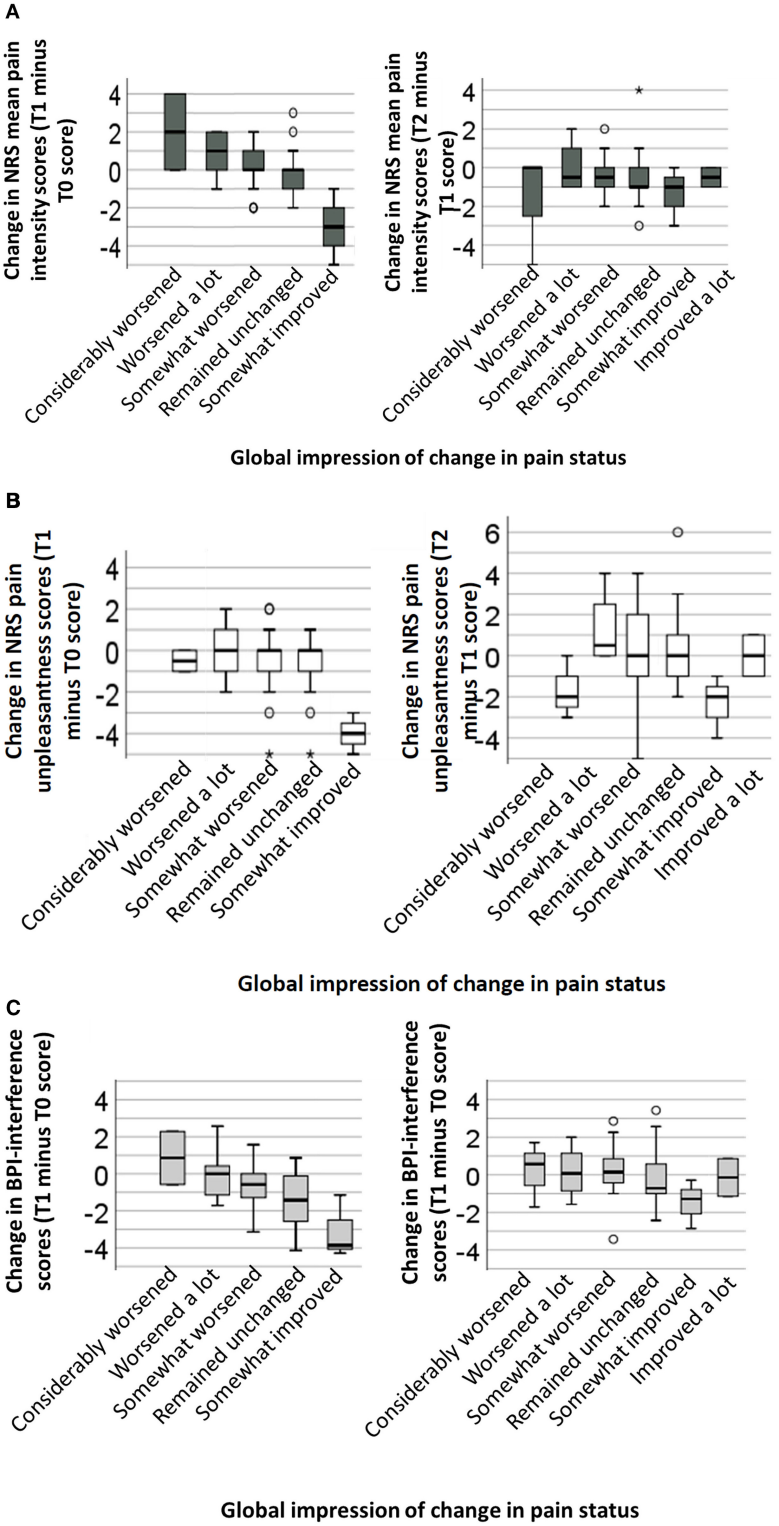
Box plots of pain changes between the first wave of the pandemic (T1) and pre-pandemic pain levels (left graph) and between the end of the first wave (T2) and during the first wave (T1) of the pandemic (right graph). Row **(A)** represents changes in pain intensity scores, row **(B)** represents changes in pain unpleasantness scores, and row **(C)** represents changes in pain interference scores. Each box represents the first (Q1) and third (Q3) quartile and the middle line represent the median. The whiskers represent the minimum and maximum (Q1 or Q3–1.5*interquartile range) of the score distribution, with circles representing outliers. A score above zero on the y-axis indicates an increase in pain/interference scores (i.e., pain deterioration) from baseline to T1 (left graph) or from T1 to T2 (right graph), while a score below zero on the y-axis represents a decrease in pain/interference (i.e., pain relief). The x-axis represents individuals' global impression of change in pain status.

In Figure 2, boxplots are displayed that show the differences in participants' report of pain intensity scores at the different time points and their corresponding reports of pain status change based on participants' global impression of change in their pain status.

*Obj 2. Baseline stress characteristics associated with evolution of pain and psychological distress*. Results of the linear mixed effects models are shown in Table 3. Higher levels of pain catastrophizing (β = 0.04, *p* = 0.028) at baseline were associated with higher pain intensity levels throughout the pandemic. Higher levels of pain catastrophizing (β = 0.05, *p* = 0.031) and perceived stress (β = 0.07, *p* = 0.048), and lower degree of vulnerability to perceived social-evaluative threat (β = −0.08, *p* = 0.032) were associated with higher levels of pain unpleasantness throughout the pandemic. Higher levels of perceived stress (β = 0.10, *p* = 0.012) and lower degree of vulnerability to unpredictability (β = −0.09, *p* = 0.037) were associated with higher levels of pain interference throughout the pandemic. Finally, higher levels of baseline perceived stress (β = 0.14, *p* = 0.002) and pain catastrophizing (β = 0.09, *p* < 0.001) were associated with higher levels of psychological distress throughout the pandemic.

**Table 3:** Linear mixed effects models examining the within-person evolution of pain and psychological distress taking into account baseline stress characteristics (*N* = 49).

**Fixed effects**	**β**	**SE**	***t***	***p***
**Pain intensity**				
Intercept	4.50	0.88	5.07	<0.001
Time	−0.10	0.12	−0.90	0.370
SCQ.control	−0.004	0.04	−0.11	0.913
SCQ.unpred	−0.02	0.03	−0.62	0.544
SCQ.ego	−0.44	0.03	−1.53	0.135
SCQ.new	0.59	0.03	1.98	0.056
PSS	0.58	0.03	2.00	0.053
**PCS**	**0.04**	**0.02**	**2.30**	**0.028**
**Pain unpleasantness**				
Intercept	6.48	1.13	5.74	<0.001
**Time**	**−0.56**	**0.15**	**−3.86**	** <0.001**
SCQ.control	0.003	0.05	0.07	0.942
SCQ.unpred	0.003	0.04	0.08	0.941
**SCQ.ego**	**−0.08**	**0.04**	**−2.21**	**0.032**
SCQ.new	0.04	0.04	0.96	0.342
**PSS**	**0.07**	**0.04**	**2.04**	**0.048**
**PCS**	**0.05**	**0.02**	**2.23**	**0.031**
**Pain interference (BPI)**				
Intercept	4.10	1.30	3.16	0.002
Time	−1.91	0.76	−2.54	0.015
Time^2^	0.37	0.19	1.92	0.060
SCQ.control	0.08	0.05	1.70	0.097
**SCQ.unpred**	**−0.09**	**0.04**	**−2.15**	**0.037**
SCQ.ego	0.02	0.04	0.45	0.655
SCQ.new	<0.001	0.04	−0.002	0.998
**PSS**	**0.10**	**0.04**	**2.62**	**0.012**
PCS	0.04	0.02	1.76	0.086
**Psychological distress (PHQ)**				
Intercept	0.11	1.30	0.09	0.933
Time	−0.08	0.16	−0.52	0.603
SCQ.control	−0.01	0.05	−0.15	0.882
SCQ.unpred	−0.03	0.05	−0.51	0.611
SCQ.ego	0.003	0.04	0.08	0.938
SCQ.new	0.04	0.04	0.95	0.348
**PSS**	**0.14**	**0.04**	**3.39**	**0.002**
**PCS**	**0.09**	**0.02**	**3.82**	** <0.001**

*B, unstandardized regression coefficients; SE, standard error; BPI, Brief Pain Inventory; PHQ, Patient Health Questionnaire-4; SCQ.control, control subscale of the Stress Characteristics Questionnaire; SCQ.unpred, unpredictability subscale of the Stress Characteristics Questionnaire; SCQ.ego, threat to the ego subscale of the Stress Characteristics Questionnaire; SCQ.new, novelty subscale of the Stress Characteristics Questionnaire; PSS, Perceived Stress Scale-4; PCS, Pain Catastrophizing Scale. Bold values indicate p < 0.05*.

Sensibility analyses did not show any significant effects of the individual STUN components (when examined in a model without the PCS and PSS or in models where SCQ subscales were examined individually with the PCS and PSS), all *p* > 0.05.

### Discussion Study 1

This study has investigated the experience of pain and stress among individuals living with chronic pain during the COVID-19 pandemic. Pain intensity scores on the NRS for the overall sample varied by <10% throughout the pandemic and pain unpleasantness and pain interference scores have improved. However, two-thirds of individuals reported that their pain status deteriorated during its first wave using the PGIC scale. Many studies report a high degree of concordance in individuals' pain intensity ratings and global impressions of change (39). However, a study of patients recruited from multidisciplinary pain clinics showed an overall subjective deterioration in pain but failed to show a significant difference in pain intensity ratings before and during the pandemic (40). Such discrepancy between pain scores and global impression of change in pain experience likely reflects the multidimensional and complex nature of the pain experience that goes beyond its intensity.

Stress was identified by more than half of participants with deteriorated pain as an important contributor to changes in pain status during the pandemic. The present study showed that individuals' tendency to ruminate, feel helpless, and magnify their pain experience, and those with higher levels of perceived stress are more likely to report higher levels of pain and psychological distress throughout the pandemic compared to those reporting lower levels at baseline.

Study results also show a small protective effect of social-evaluative threat on pain unpleasantness. This might be because the pandemic had sheltered us from social interactions and indirectly decreased the likelihood of encountering events that pose a social-evaluative threat. As such, those individuals most vulnerable to this type of stress experienced the largest benefits on pain unpleasantness. In addition, study results showed a small protective effect of sensitivity to unpredictable events on levels of pain interference. It is possible that those vulnerable to unpredictable situation react to this vulnerability by being more proactive in their environment in an attempt to reduce as much as possible sources of uncertainties. This attitude might in turn lead to increased levels of engagement in daily activities and thus reducing pain interference.

Global and multifactorial measures of stress (PCS and PSS) seem to have a stronger impact on pain outcomes however, compared to individual components of the STUN model. Perhaps given the magnitude of the pandemic, a global measure that captures many dimensions of stress would capture more variance in pain outcomes compared to individual components of the STUN framework. Many scales are now available to measure stress specifically in the context of the pandemic, such as the COVID Stress Scales (41) and the COVID-19 Phobia Scale (42).

## Study 2—Qualitative Exploration of Stress and Pain During the Pandemic

### Material and Methods

#### Design of the QUAL Study

Semi- structured one-on-one interviews were carried out between March and May 2020 to explore the associations between stress and chronic pain in a pandemic context among individuals living with chronic pain. These individuals were recruited among a sample of 41 individuals who had participated in a focus group about stress and pain in 2019 (22). Given the different objectives of these two phases and content of the interview guides, these data are not analyzed jointly and here we focus only on the semi-structured interview data.

#### Participants

Out of 41 eligible individuals, 32 participants (16 women and 16 men) were randomly contacted by phone to inform them of the project until optimal sample size was achieved. Twenty-seven participants agreed to take part in an online interview and provided written consent electronically. Those participants were 18 years of age or older and living in the province of Quebec, fluent in spoken French, and living with chronic pain (>3 months) of moderate to severe intensity (>3/10). Final sample size was determined based on a number of factors, including timeline (interviews had to be conducted over the shortest time period possible in order to have the most homogeneous public health restrictions in place when participants were interviewed) (43) and methodological considerations for thematic analysis, including data saturation and thematic prevalence (44), narrow study aim, moderate sample specificity, and case analysis strategy (45).

Characteristics of participants involved in this qualitative part of the study are shown in Table 4. Information on participants' ethno-racial background was not collected in this study. None of the participants had been diagnosed with COVID-19 but 3 reported symptoms at the time of the interview.

**Table 4:** Participant characteristics of the qualitative study (*N* = 27).

**Variables**	***N* (%)**
**Sex**	
Males	12 (44.4%)
Females	15 (55.6%)
**Age range**	
<40 years	4 (14.8%)
40–69 years	18 (66.7%)
>70 years	5 (18.5)
**Education level**	
High school or less	0 (0%)
College or technical degree	12 (44.4%)
University	15 (55.6%)
**Exposure to the COVID-19**	
Diagnosed with the COVID-19	0 (0%)
Currently presenting symptoms of the COVID-19	3 (11.1%)
Been in contact with someone diagnosed with the COVID-19	1 (3.7%)
**Pain duration**	
0–2 years	1 (3.7%)
3–5 years	3 (11.1%)
6–10 years	4 (14.8%)
11–20 years	7 (25.9%)
21–30 years	8 (29.6%)
>30 years	4 (14.8%)
**0–10 pain intensity (original study—2019)**	
4-6	19 (70.4%)
≥7	8 (29.6%)
**0–10 pain intensity (phase 2—2020)**	
0–3	5 (18.5%)
4–6	13 (48.1%)
≥7	9 (33.4%)
**Public health safety measures that directly impacted participants**	
Dependent children at home	5 (18.5%)
Remote work	3 (11.1%)
Temporary loss of employment	3 (11.1%)
Canceled medical appointments	18 (66.7%)
Decreased medical assistance	9 (33.3%)
Reduction in assistance received from relatives	9 (33.3%)
Restrictions on leaving home (e.g., >70 years old, immunocompromised)	12 (44.4%)
Voluntary 14-day confinement	13 (48.1%)

#### Procedure

Participants completed a sociodemographic questionnaire online prior to engaging in an individual interview online via the platform Zoom that lasted between 30 and 80 min. Interviews were conducted using a semi-structured guide. Interview topics included overall stress experience in the context of the pandemic, the impact of stress related to the pandemic on their pain condition and its treatment and management and coping with stress and pain during the pandemic. Conversations were audio-recorded and transcribed verbatim.

Interviews were conducted by one of two interviewers (MP or ÉD). MP is a female clinical psychologist and pain researcher trained in qualitative and mixed methods. ÉD is a female sociologist trained in qualitative research. Participants were informed about the study goals, i.e., to revisit the relationship between stress and pain but this time in the context of the COVID-19 pandemic. All interviews were conducted in French and data analysis was also conducted in that language in line with recommendations for qualitative analysis and result dissemination in a different language than the one of data collection (46). Final themes and selected quotes were translated into English by a professional translator.

#### Data Analysis

Reflexive thematic analysis was used as the primary data analysis method, using patterns of shared meaning (47, 48). An inductive approach was mainly used to explore specifically characteristics of stress and pain present in the data. Contextualization of these characteristics within the broader lived experience of participants in the context of the COVID-19 pandemic was then explored. While the STUN framework helped to interpret results from the analysis, it was not used to identify theme or classify types of stress experienced by participants. Attempts were made however, to evaluate whether the STUN characteristics are relevant to the experience of stress during the pandemic.

The lead analyst and two other team members established a preliminary and evolving codebook (49); frequent meetings were held to arrive at a common codebook. Process and open codes through a line-by-line analysis were used to move toward an interpretive level of analysis and the generation of themes (50). An iterative approach moving several times between raw data and ongoing interpretation and reflections on participants' experiences was used. Several team meetings took place to construct themes. Member checking and audit trails were used to enhance trustworthiness of the data (51). NVivo-12 (52) was used to code data into domain summaries.

### Qualitative Results (QUAL)

Data analysis aimed to explore the dynamic impact of stress on the pain experience during the first wave of the COVID-19 pandemic. The experiences of participants were heterogeneous, with some reporting little to no impact of the pandemic on their stress, pain or daily routine, while others described feeling heavily the bidirectional effects of the pandemic and pain conditions. Five themes were identified: (1) status quo: between philosophy and stability of life and health stages; (2) pain management in socially exposed and disrupted environments; (3) further complicating access to pain care: adding insult to injury; (4) avoidance as a stress response to an invisible threat; and (5) silver lining: regaining control of pain during an uncontrollable pandemic.

#### Status Quo: Between Philosophy and Stability of Life and Health Stages

A few participants reported minimal disruptions to their daily life during the ongoing pandemic. These individuals described having well-established pain care plans that were not disrupted by the lockdown measures, or their work and social statuses were less likely to be disrupted because they were retired for example.

“How is my stress… well yesterday we baked bread! (laughs) And so we don't have food problems anymore. And me and my spouse we have been married for 46 years, so we get along very well…. We talk, we do things together…. Things are going well. And on top of that, there are less people coming over for dinner! And we don't go to other people's places for diner! So there is less going back and forth and that suits me very well!” (P.13, M, 64 years old)

Beyond the stability of one's life and health stage, one's philosophy also helped minimize the impact of the pandemic on their pain and stress levels. Those individuals tended to focus on aspects that they could control, and on the present moment. By doing so they were able to reduce the perceived lack of control and unpredictability of the pandemic.

“I often say that nowadays: I don't accept my pain, but I'm learning to live with it. It'll be the same thing with the pandemic. I don't accept the virus, but I've got no choice but to learn to live with it! And learn to live differently! It's the same as with my pain!” (P.11, M, 78 years old)

For others however, the absence of added stress from the pandemic came rather from the perspective that one's situation was already so poor that it could not further deteriorate.

“The pandemic hasn't had any particular impact, because I was already all destroyed, or almost. This is normal as health problems like this one play out. It can go as far as social isolation! You can no longer have a social life with people in the same way!” (P.20, M, 58 years old)

#### Pain Management in Socially Exposed and Disrupted Environments

Managing pain during a pandemic was difficult for many participants who struggled with the increased cognitive and physical workload brought on by the pandemic in their personal and/or professional lives. This translated for some into increased difficulties to apply pain management strategies.

“[My pain] has completely increased… It is hard to manage right now. We have so many other things to manage, other things to think about. As stress increases, pain management becomes more difficult.” (P.6, F, 34 years old)

Furthermore, the altered social environment, such as all family members suddenly staying home, exposed people's pain to broad daylight, making it much more challenging to hide it. This confinement decreased their ability to manage pain and hide it from others, thus threatening their ego.

“Then, on top of it, they see the pain I've got. Normally they don't see it so much. They'd see it in the evening, but now when you're with somebody all day long and then the person… you see that they're in pain all the time. [.] It's like showing your family another part of the pain, so it's harder.” (P.6, F, 34 years old)

The constant tension between needing to engage in self-care or pain management and caring for the needs of others (e.g., having to care for children who are at home) was highly stressful and led to a vicious cycle of increased pain that then fed their stress. This was particularly discussed by younger participants. At times, this vicious cycle led to an under-utilization of non-pharmacological means to manage pain and increased pain medication intake.

“Before, since I was all alone during the day, I'd take a nap and often in the afternoon I'd feel better… I was able to do things to reduce the pain. And now, since the kids are here all the time and they're asking for things, they keep asking, asking!. So, I just can't manage my pain like I used to. Now I'm managing my kids. So, my pain, I manage it more with meds now.” (P.12, F, 39 years old)

#### Further Complicating Access to Pain Care: Adding Insult to Injury

Many participants feared that unstable access to pain management because of postponed or canceled appointments would lead (or had already led) to significant deterioration of their pain condition. This was also the case for physical and psychological pain management strategies. In this case, the threat was not only a worsening of their pain condition, but also of their social and psychological well-being.

“When they closed the gyms that was a big deal for me. Because it's the only physical exercise I can do. It's good for the. it allows circulation in what I've got left. I'm afraid I'm going to lose this arm [the right arm] at some point. This arm [the left arm] is starting to get cold. I'm afraid that algodystrophy will get into it. It's a bit of a drag. Going to the gym has psychological benefits. It's like you took away my pub, by doing that. They've taken away my social club. By closing the gym, they took a lot away from me. It's a big deal.” (P.18, M 57 years old)

During the pandemic, there has been new solutions, such as online care and activities, to provide social interactions and meet the self-care needs of the general population. However, for those who needed health care services, these solutions seemed to increase one's frustration and stress.

“We're told: be creative! Do some meditation at home. Do some painting, and all the rest. It's all very well to do some painting. Instead of just making life livable for that person, to say: to forget your misery, you can… make paper-maché sculptures! Well I don't want paper-maché, I want massage therapy. They make me do paper-maché to help me forget that I don't have any services, that my life is just poop. But (laughs) at one point, it's NO! That's enough!” (P.27, F, 48 years old)

#### Avoidance as a Stress Response to an Invisible Threat

The virus posed an invisible threat for many individuals who perceived themselves as being at higher risks of dying should they get infected. Many individuals perceived their health as fragile in part because of chronic pain.

“I already have so much difficulties trying to be the woman I used to be. I will never be that woman again. But if I catch [the virus] on top of it, I don't think I'll be able to get through this. Just sometimes I cough let‘s say because I have a dry throat. It pulls, it really hurts, I am writhing in pain. If I should catch something like this virus, I won't survive.” (P.6, F, 34 years old)

The novelty of the virus and lack of knowledge about modes of transmissions, the unpredictability of one's chance of surviving if they get infected, and the lack of control over the situation were important sources of stress. This perceived threat had a significant impact on their behaviors, including increased hesitation at seeking medical care for pain.

“I would not want to end up in the hospital… We don't want pain to increase, but we don't want to end up in the health care system for COVID-19.” (P.21, M, 59 years old)

#### Silver Lining: Regaining Control of Pain During an Uncontrollable Pandemic

The stability of one's pain condition and ongoing treatment prior to the COVID-19 pandemic had a large impact on an individuals' stress appraisal of the pandemic. For some, the pandemic had positive impacts on their pain management opportunities by providing them with more time to devote to their pain care.

“And I take advantage of it because I'm slower in my personal activities. So, I take at least two breaks each day, for my treatments [TENS]. And I can only have this treatment when the pain isn't too intense, because when it is, I can't take these electric shocks.” (P3, M, 78 years old)

This was also the case for those who perceived the pandemic as a break from having to push the limits of their physical capacities in order to meet their basic needs and as a temporary protection against the threat of pain on one's sense of identity.

“It's less confrontational not to have to do something than it is to have to do it and tell you, well, right now I'm dragging a 40-pound load, the stairs in the subway are out of order, I have two huge landings, and no one's stopping to help me… So now it's more like “don't take public transit!” So now what you're doing to me, is that I don't have to suffer, and I don't have to be humiliated? Cool!” (P27, F, 48 years old)

### Discussion Study 2

This study explored the dynamic impact of stress on the pain experience during the first wave of the COVID-19 pandemic. Stress and pain responses to the pandemic were heterogeneous and seemed influenced by many factors, such as one's life stage and social situation, pain condition and stability of ongoing treatments, degree of precariousness, and level of adaptability. While many reported negative impacts of the pandemic on their pain and overall well-being, others perceived opportunities to further adapt their pain management strategies or focus on elements that were within their control to minimize stress.

As mentioned previously, the STUN framework identifies four specific characteristics of situations that will trigger a physiological stress response: novelty, unpredictability, threat to the ego and sense of low control (19–21). Many of these characteristics could be observed in participants' narratives. Those perceiving having little control over an unpredictable pandemic, pain condition, and access to pain care often felt overwhelmed, stressed and with increased pain levels. Few participants described that focusing on aspects over which they had control, such as strictly following public health safety recommendations, served as a buffer against potentially stressful situations. Threat to the ego was also discussed in the context of increased in-home social interactions, making it more difficult to hide their pain. For others however, this social threat of pain was decreased because they either lived alone or had significantly less interactions with the outer world. Among the four characteristics of the STUN framework, the novelty was the least discussed. This might be in part because chronic pain requires one to constantly navigate new challenges and as such the pandemic wasn‘t such a departure from their constant need for adaptation. This could also be in part because this pandemic was a novel experience for most of the world population and as such wasn't discussed specifically in the interviews focused on living with chronic pain.

## Mixed Methods Integration

Considering the quantitative findings, the mixed methods objective was to better understand how individuals' pain condition evolved throughout the first wave of the pandemic, and the extent to which stress played a role in this evolution. The integration process was particularly focused on obtaining a deeper understanding of the incongruent pain status and pain intensity reports in the quantitative finding, and how stress and other dimensions of pain could help better understand individual experiences. This was done by merging to two databases for analysis.

### Methodology for Mixed Methods Integration

The quantitative data was given more weight in the integration, and qualitative data was mainly used to elucidate puzzling quantitative findings regarding discrepancies between scores on the global impression of change in pain status and pain intensity. The databases were examined contiguously, and then together to compare and contrast findings using primarily joint displays (53–55). Codes and themes obtained from the qualitative analysis were examined in the context of the quantitative results. More specifically, three frequently occurring types of quantitative profiles characterizing changes associated with the pandemic were identified in the data using a cross-tabulation: global impression of change worsened and pain increased (NRS score); global impression of change worsened and pain decreased; and global impression of change unchanged/improved and pain decreased. Then, participants of the qualitative study were also categorized based on change in their NRS pain score obtained before each interview as increased, unchanged or decreased pain intensity score before and during the first wave of the pandemic. Their qualitative data was then coded to capture their subjective impression on the evolution of their pain during the pandemic (e.g., how participants described their pain evolution since the beginning of the pandemic). This allowed to explore whether the quantitative profiles found in Study 1 were also present in Study 2 and whether other profiles could also be identified. This structure allows for exploration of conceptual similarities between the variables and themes and how they interact (55). This data integration was presented in the form of joint displays, which are visual integration of quantitative and qualitative findings that aim to generate new insights.

### Integration of QUAN and QUAL Findings

**Figure 3:**
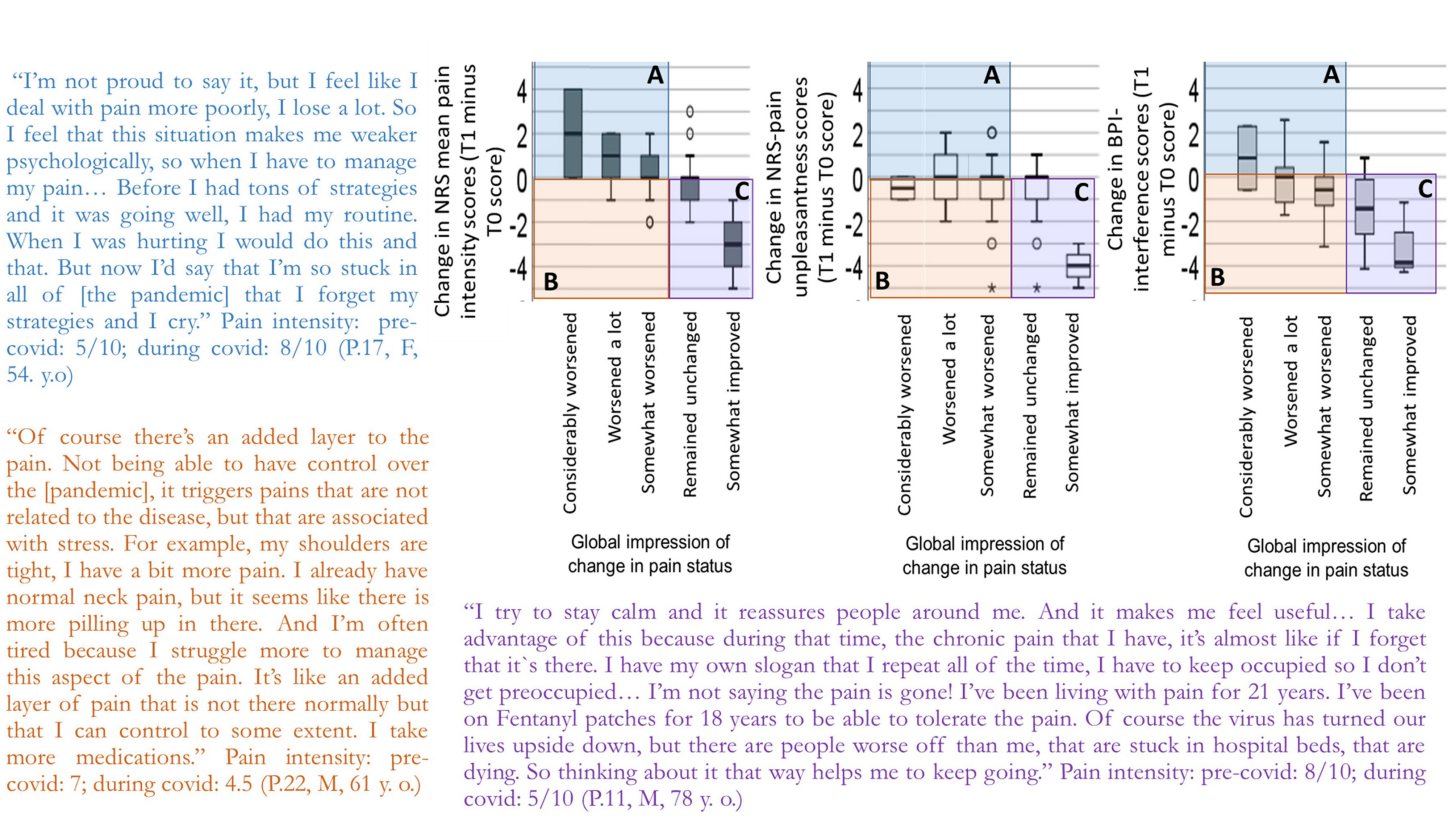
Quantitative reports of pain intensity (left graph), pain unpleasantness (middle graph) and pain interference (right graph) scores and global impression in pain status from the quantitative study and citations of qualitative study participants. For each graph, the x-axis represents individuals' global impression of change in pain status. The y-axis represents the difference in pain intensity, unpleasantness or interference scores from T1 minus T0. **(A)** Pain worsened consistent with a deterioration in the global impression of change in pain status. This was the case for 26 individuals for pain intensity, 18 individuals for pain unpleasantness, and 12 individuals for pain interference. **(B)** Pain improved but the global impression of change in pain status suggests a general deterioration reflecting other pain/stress-related factors. This was the case for 6 individuals for pain intensity, 14 individuals for pain unpleasantness, and 20 individuals for pain interference. **(C)** Pain improved and global impression of change in pain status suggests stability or some improvement. This was the case for 7 individuals for pain intensity, 9 individuals for pain unpleasantness, and 13 individuals for pain interference. The qualitative study allowed to identify similar profiles of participants that provide context to those ratings.

Given that participants in the qualitative study were recruited from an earlier focus group study, their individual pain scores pre-pandemic were available and examined (see Table 4). Specific profiles of narratives offering a deeper understanding of the quantitative pain intensity and global pain status ratings are shown in the joint display in Figure 3. This display highlights the presence of three distinct profiles of individuals who participated in the quantitative study. The profiles were derived using a cross-tabulation using the change in NRS pain score from the first wave of the pandemic compared to their pre-pandemic score, and their global impression of change score related to their pain status since the beginning of the pandemic. The first profile represents individuals who report a worsened pain status on the PGIC and report an increased pain on the NRS-11 from pre- to during the pandemic. The second profile represents individuals who also report a worsened pain status on the PGIC but report a decreased pain on the NRS-11 from pre- to during the pandemic. Last, the third profile represents individuals who report an unchanged or improved pain status on the PGIC while reporting a decreased pain on the NRS-11 from pre- to during the pandemic. No individuals reported an unchanged/improved pain one the PIGC while reporting a deteriorated pain on the NRS-11 from pre- to during the pandemic. Participants in the qualitative study were also categorized based on their NRS-11 scores as having deteriorated or improved/unchanged. Their narrative were analyzed to understand their impression of pain evolution, in order to further our understanding of the quantitative profiles.

#### Increased Pain Intensity Ratings and Global Impression of Pain Deterioration

As shown in quadrant A in Figure 3, many individuals reported coherent ratings of pain deterioration in the measure of global impression of change combined with increased pain scores, reflecting struggles to adapt to the pandemic in terms of both stress and pain. The intensity of their pain itself increased, and this is to be understood in the context of a global deterioration of their physical condition, well-being, and often social environments.

#### Decreased Pain Intensity Ratings and Global Impression of Pain Deterioration

As shown in quadrant B in Figure 3, a few participants, despite reporting a decreased pain intensity score from baseline to the first wave of the pandemic, reported that their pain condition had deteriorated. Pain being a biopsychosocial experience, degradation of the psychological and social components of pain negatively impacted individuals' perception of their overall pain condition. For example, the fact that others were less emotionally available, having less access to a social network, increased level of suffering, and uncertainty about the resolution of the turmoil produced by the pandemic were discussed as being embedded in their overall pain experience.

#### Decreased Pain Intensity Ratings and Unchanged/Improved Global Impression of Change

As shown in quadrant C in Figure 3, approximately one-third of individuals in the quantitative study reported no change or in few instances an improvement of their pain condition and reported decreased pain intensity ratings during the first wave of the pandemic compared to baseline. These individuals tended to have relatively stable life circumstances (e.g., not being in the workforce), living with a partner, having a stable source of income, and having a well-established pain care plan unaffected by the lockdown measures. Adopting an empathic stance toward those affected more directly by the pandemic helped decrease the social threat posed by such novel event and turned the focus away from pain.

## Discussion

This research has investigated the experience of pain and stress among individuals living with chronic pain during the COVID-19 pandemic using a mixed methods approach. Several key findings emerged from this research.

### Levels of Stress and Pain During the Pandemic

A significant proportion of individuals in this study reported a deterioration of their pain condition during the first wave of the pandemic, in agreement with another Canadian study (10). Results of linear models exploring pain scores over time showed however no change, or slight improvement in the case of pain unpleasantness and pain interference over time. This would be consistent with studies from the United States and Europe which found that most participants reported unchanged pain severity at the beginning of the pandemic (56, 57). One has to be careful when comparing data across countries, given differences in the local state of the pandemic during data collection, strictness of lockdown measures in place, and extent of disruption of the health care system. Nonetheless all studies identified subgroups of individuals who faired relatively well during the pandemic. As documented in another qualitative Canadian study, some individuals perceived an improved quality of life during the pandemic, either because the world had slowed down to a pace that is more compatible with their level of functioning (e.g., decreased requests for social outings) or because they had more time to focus on pain management (58). It is also possible that some individuals with chronic pain have developed a resilience to overcome challenges and obstacles and a flexibility to engage in new or alternative pain management strategies. Psychological flexibility has been identify in another study as an important contributor to individuals with chronic pain' psychological well-being and pain interference during the pandemic (59). The concept of resilience as facilitating adaptation to chronic pain has also been documented in other contexts (60).

Many studies have documented that stress is common (61–65). In the pain literature, while levels of stress are also generally more elevated during the pandemic, there is controversy regarding whether this increased stress leads to worsened pain (66, 67). Not all were equal in the face of stress, however. In the present study, those whose daily routine were disrupted or were in more precarious socioeconomic situations were more likely to face multiple stressors due to the pandemic. This is consistent with a European study that found that levels of economic vulnerability increased one's risk of experiencing anxiety, depression, and stress during the lockdown to control the spread of COVID-19 (68).

### Multidimensional Impact of Stress on Pain

Sources of stress were numerous and diverse during the pandemic. For individuals living with chronic pain, this included environmental stressors of the pandemic itself (exposure to the virus, lockdown measures), but also pain-specific stressors (e.g., postponement of medical appointments, decreased help from others) (59, 69). Given individuals' vulnerabilities to stress, social context and pain condition, the impact of the pandemic on their pain journey was heterogeneous (58, 70, 71). Pain appeared to be affected by stress in multiple ways, including overwhelming cognitive load that made it more difficult to engage in pain management, decreased social contexts conducive to pain management, anxiety, fatigue and apathy that decreases one's ability to cope with pain (57). Given the observed heterogeneity in participants' contextual factors (e.g., stability of pain treatments, socioeconomic status, social support), the association between stress during the pandemic and pain outcomes remains complex and multifactorial.

### Global Perceived Stress vs. Individual Components of the STUN Framework

Quantitative and qualitative studies identified individual components of the STUN framework associated with participants' experience of the pandemic and its impact on pain. For example, lack of control over and unpredictability of the pandemic and pain dynamics led some participants overwhelmed and feeling vulnerable to the escalation of both stress and pain. For others, focusing on controllable aspects of their day-to-day life seemed to decrease their levels of stress. Perceived control over time was identified in one study as an important factor associated with anxiety and fear of COVID-19 pandemic (72). The unpredictable evolution of the pandemic and its overall stress load were also identified as important determinants of burnout syndromes in different populations, such as healthcare workers (73). Quantitative results, however, suggest that general measures of stress and pain catastrophizing before the pandemic are associated with pain dimensions during the first wave of the pandemic, more so that one's vulnerability to individual components of the STUN model. This might be because the pandemic at is onset disrupts so many aspects of individuals' lives, including work, social relations, health care behaviors, and survival that not one single component will capture all these facets. As individuals learn to live in a pandemic and as specific pandemic-related issues emerge (e.g., polarization of opinions on confinement measures or vaccines), specific characteristics of the STUN model (e.g., social-evaluative threat) would have a larger influence on individuals' experiences.

## Strengths and Limitations

One important strength of this study is the capture of stress and pain data at baseline, before the beginning of the pandemic. This provided a rare opportunity to explore how stress, pain and their associations evolved during and after a natural world-wide stress exposure. The use of mixed methods also added value to both quantitative and qualitative findings and provided new insights that would not have been possible without data integration. Nonetheless this study also has some limitations. The sample size of the quantitative component is relatively small, but it was not possible to increase sample size once the state of emergency had been declared in Canada due to its influence on baseline stress data. As a result, the number of independent variables examined was limited. The small sample size might have also introduced a selection bias, and limits generalizability of study findings to different chronic pain populations. Participants were recruited from a single province, namely the one reporting the highest number of COVID-19 cases during the first wave of the pandemic. As such, results might not be generalizable to individuals from other provinces or other countries. In addition, the level of education, particularly in the qualitative sample was high and might not reflect the situation of many individuals living with chronic pain. Also, some study questions, such as the Stress Characteristics Questionnaire, do not have published data on their psychometric properties and as a result their validity and reliability have not yet been demonstrated. Finally, both samples had socioeconomic diversity but lacked in ethnic diversity with participants being predominantly White in study 1 and this information was not captured in study 2.

## Conclusions

Multiple sources of stress associated with the COVID-19 pandemic were identified among individuals with chronic pain. While some participants reported little impact of the pandemic on their stress and pain status, most identified significant difficulties in managing pain and stress in this context. For future COVID-19 waves and pandemics, it will be crucial to develop interventions (e.g., individual and/or family programs aimed at optimizing well-being, stress and pain management in the context of shifted routines and roles) and community support (e.g., programs adapted to the specific challenges faced during the pandemic) that are tailored to the needs and physical capacities of individuals living with chronic pain.

## Data Availability Statement

The data that support the findings of this study are available from the corresponding author, A. Lacasse, upon reasonable request and conditionally to a proper ethical approval for a secondary data analysis. The data are not publicly available since participants did not initially provide consent to open data.

## Ethics Statement

The studies involving human participants were reviewed and approved by Comité d'éthique à la recherche du Center hospitalier de l'Université de Montréal. The patients/participants provided their written informed consent to participate in this study.

## Author Contributions

MP, MR, PR, ÉV-P, and ÉD were involved in study design. MP and ÉD collected data. MP, ÉD, and LD were the primary authors involved in the qualitative analysis and mixed methods integration. Results were discussed with all study authors and subsequently refined. All authors contributed to the writing and/or revisions of the manuscripts and all approved its final version.

## Funding

This study was funded by a stimulus grant awarded to MP from the Quebec Pain Research Network, a network funded by the Fonds de recherche du Québec-santé. MP and ÉV-P are Junior 1 research scholars from the *Fonds de recherche du Québec-santé*. MR holds a Canada Research Chair in Brain Imaging of Experimental and Chronic pain. SL holds a Canada Research Chair on Human Stress.

## Conflict of Interest

MP received honoraria as a speaker for Canopy Growth for content unrelated to the work presented here. The remaining authors declare that the research was conducted in the absence of any commercial or financial relationships that could be construed as a potential conflict of interest.

## Publisher's Note

All claims expressed in this article are solely those of the authors and do not necessarily represent those of their affiliated organizations, or those of the publisher, the editors and the reviewers. Any product that may be evaluated in this article, or claim that may be made by its manufacturer, is not guaranteed or endorsed by the publisher.
