# Supplementary material for: Stress and Pain Before, During and After the First Wave of the COVID-19 Pandemic: An Exploratory Longitudinal Mixed Methods Study
Source: Front Pain Res (Lausanne). 2021 Nov 24;2:725893. doi: 10.3389/fpain.2021.725893 (PMC8915720; doi:10.3389/fpain.2021.725893)
Supplement: Supplementary file 1 [file Data_Sheet_1.docx]

**Semi-structured interview – stress-pain/covid-19**

The purpose of this interview today is to understand the impact of the COVID-19 pandemic on your experience of stress and pain. A few months ago, we explored the links between stress and pain in a group interview with you and other individuals living with chronic pain. We would like to revisit these experiences taking into account the particular context that we are all currently experiencing.

1. **First, tell me about your stress levels over the past few days.**
   1. What are the main sources of stress you are currently experiencing?
   2. What might be stressful about the coronavirus? Or the restrictions imposed to reduce the transmission of the virus?
2. **Those situations can be difficult for many individuals? I would like us to reflect on the elements that make these situations you have just described stressful.**
   1. How do you feel when you experience these situations?
3. **How do you manage this stress?**
   - 1. Are there things that make it easier to manage this stress?
     2. Are there things that make it more difficult to manage this stress?
4. **How does this stress affect your pain?**
   1. What impact does this stress have on your pain intensity, quality, level of functioning??
   2. What impact does this stress have on your emotions related to pain (unpleasant nature of pain for example)?
   3. What impact does this stress have on pain-related suffering?
   4. How does this stress impact your pain management?
5. **Conversely, how do you think pain is affecting your experience of the COVID-19 pandemic?**
   1. How does pain influence how vulnerable you feel about the impacts of the pandemic?
6. **Last summer we discussed the link between stress and pain. How does the stress associated with the COVID-19 pandemic change this link?**
   1. Impact on its management
7. **We also talked about the suffering that was associated with pain and stress. What do you have to tell me about this right now?**
   1. How does pain change in a pandemic context?
8. **Sometimes the pain itself is a source of stress. What are the stressful aspects of your pain in recent days?**
   1. What changes have you noticed in your pain over the past few days/weeks?
   2. Could you describe the impact of your pain on your stress levels?
   3. What difficulties or improvements do you anticipate in managing your pain in the coming weeks?
   4. What difficulties or improvements do you anticipate in accessing adequate treatments for your pain in the coming weeks?
9. **I would like now to discuss the social aspects of the COVID-19 pandemic and the impact on your pain. How would you describe the impacts on your social life?**
   1. What are the impacts of the pandemic on your social life?
   2. How do these changes in your social life affect you?
   3. How are they influencing your mood?
   4. How do you manage these changes?
10. **Do you have any ideas of what could be put in place to help people living with chronic pain during the COVID-19 pandemic?**
    1. What are the things you would need to reduce its impact?
    2. What things have you put in place that are currently helping you?
