# Supplementary figures and images for: Stress and Pain Before, During and After the First Wave of the COVID-19 Pandemic: An Exploratory Longitudinal Mixed Methods Study
Source: Front Pain Res (Lausanne). 2021 Nov 24;2:725893. doi: 10.3389/fpain.2021.725893 (PMC8915720; doi:10.3389/fpain.2021.725893)

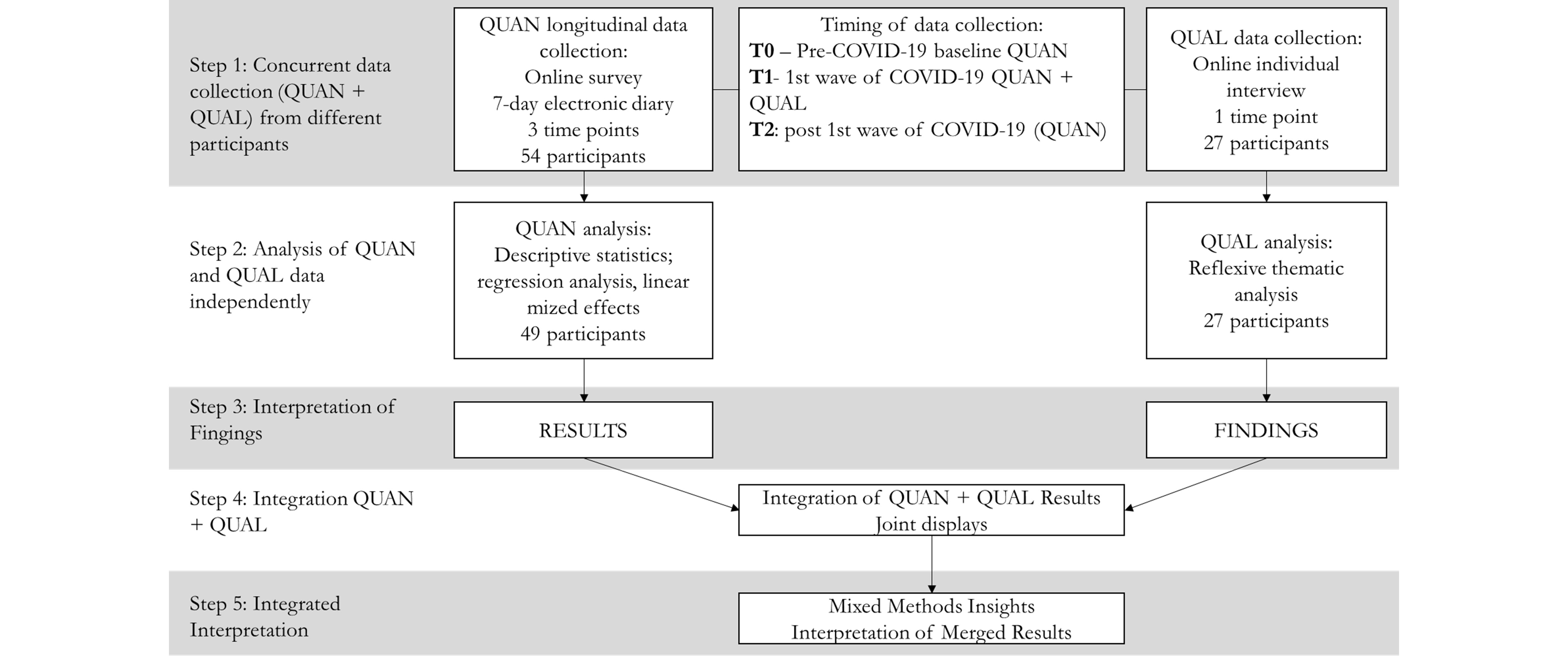

Supplement: Supplementary file 2 [file Image_1.JPEG]
